# Influence of Tooth Age On Intracanal Dentin Adhesion

**DOI:** 10.3290/j.jad.c_1980

**Published:** 2025-04-30

**Authors:** Jacqueline Victoria Krempels, Richard Sturm, Konrad Neumann, Tamara Schumacher, Christian Schouten, Franz-Josef Faber, Roland Frankenberger, Matthias Johannes Roggendorf

**Affiliations:** a Jacqueline Victoria Krempels Dentist, Department for Operative, Preventive and Pediatric Dentistry, Charité – Universitätsmedizin Berlin, Aßmannshauser Straße 4-6, 14197 Berlin, Germany. Performed bond strength experiments, wrote the manuscript.; b Richard Sturm Dentist, Department for Operative, Preventive and Pediatric Dentistry, Charité – Universitätsmedizin Berlin, Aßmannshauser Straße 4-6, 14197 Berlin, Germany. Performed statistical tests and proofread the manuscript.; c Konrad Neumann Statistician, Institute of Biometry and Clinical Epidemiology, Charité – Universitätsmedizin Berlin, Charitéplatz 1, 10117 Berlin, Germany. Performed statistical tests.; d Tamara Schumacher Dentist, Private Practice. Performed bond strength experiments.; e Christian Schouten Dentist, Private Practice. Performed bond strength experiments.; f Franz-Josef Faber Physicist, Pre-Clinical Department, University of Cologne, Kerpener Straße 32, 50931 Cologne, Germany. Research idea, designed testing assembly and contributed to discussion.; g Roland Frankenberger Professor, Policlinic for Operative Dentistry, University Dental Medicine, Philipps University Marburg and University Hospital Giessen and Marburg, Campus Marburg, Georg-Voigt-Straße 3, 35039 Marburg, Germany. Research ideas and contributed substantially to the discussion.; h Matthias Johannes Roggendorf Associate Professor, Policlinic for Operative Dentistry, University Dental Medicine, Philipps University Marburg and University Hospital Giessen and Marburg, Campus Marburg, Georg-Voigt-Straße 3, 35039 Marburg, Germany. Research idea, contributed substantially to discussion, and proofread the manuscript.

**Keywords:** luting systems, post adhesion, root canal dentin, tooth age

## Abstract

**Purpose:**

To investigate the effect of tooth age on dentin adhesion of different luting systems to the root canal.

**Materials and Methods:**

180 root canals of extracted teeth were divided into three age-specific groups (n = 60): young 20–35 (y), middle-aged 45–60 (m), and older 70–85 (o) years. Ten teeth of each age group were assigned to a luting system: Panavia 21 with ED Primer (P21, Kuraray); Core X Flow with Prime&Bond active and Self-Cure Activator (CXF, Dentsply Sirona); Multilink Automix with Multilink Primer (ML, Ivoclar Vivadent); Panavia SA Cement Plus (PSA, Kuraray); Smart Cem 2 (SM2, Dentsply Sirona); Speed CEM Plus (SCP, Ivoclar Vivadent).

The root canals of decoronated teeth were instrumented with F360 (Komet) and BR7 (FKG) up to a working length of 8 mm (Ø0.6mm, taper 0.02) and filled with standardized steel spreaders and the selected material. The intracanal bond was determined by a pull-out test. The failure modes were categorized as an adhesive to dentin (AD), adhesive to spreader (AS), cohesive within the composite (C), and mixed (M). Statistical analysis was performed using non-parametric ANOVA, Tukey, and Chi-square test at a significance level of α ≤ 0.05.

**Results:**

The study showed significant differences for the various luting systems (ANOVA, P < 0.05). PSA showed significant differences in bond strength to SM2, CXF, SCP, and ML, as did SM2 to P21 and SCP (Tukey, P < 0.05). M (46%) occurred 53% in y and 70% in SCP.

**Conclusions:**

No adhesive strategy can yet be recommended for tooth age. Clinically available luting systems show significant differences in their adhesion values.

The success of adhesive bonding in root canals is fundamentally influenced by both the chemical and physical characteristics of the luting systems and the condition of the dentinal substrate. With age, dentin undergoes natural adaptations in response to wear,^4^ making tooth age a possible influencing factor in determining adhesive performance.

Starting from the third decade of life, a process of mineralization begins in the dentinal tubules, particularly in the apical region, which progressively reduces permeability^21^ as the tubules narrow. This process has profound implications for both diagnostic and restorative dental procedures, emphasizing the need for treatments tailored to the patient’s age.^19^

In root canal adhesion,^6^ several challenges arise, such as irregular dentin structures, a reduced number of open dentinal tubules,^14^ restricted access, limited visibility, and a high C-factor.^22^ These factors compromise adhesion, making the use of less technique-sensitive adhesive luting materials highly desirable. One promising approach is the use of integrated acidic “self-etch” monomers, which simplify the bonding process by simultaneously penetrating, demineralizing, and hybridizing the dentin while bonding to hydroxyapatite, thereby reducing operator-dependent application errors.^11^

However, despite the importance of tooth age in influencing dentin adhesion, the effect of this factor on the bond strength of various adhesive strategies – one-step or multistep – is still not fully understood. This calls for further clinical research to thoroughly investigate the relationship between tooth age and intracanal dentin adhesion.^4, 10^ The aim of this study was to examine how tooth age affects the bond strength of three multistep adhesive systems and three self-adhesive one-step systems within the root canal.

The null hypothesis tested was that there were no significant differences in dentin adhesion between the various luting systems across different age groups.

By gaining deeper insights into the influence of age on root canal adhesion, this research could guide clinicians in selecting the most effective adhesive strategies tailored to the needs of patients across different life stages, ultimately improving clinical outcomes in endodontic treatments.

## MATERIALS AND METHODS

### Sample Preparation

For this study, teeth extracted for periodontal or orthodontic reasons were carefully labeled according to the age of the patients and initially examined using radiography.

A total of 180 permanent human teeth were selected based on specific criteria: each tooth featured root canals that were as straight and circular as possible, had a minimum canal length of 8 mm, were narrow, and had not undergone any prior endodontic treatment. These teeth were preserved in a moist environment within a 0.9% isotonic saline solution (NaCl) containing 0.001% sodium azide (NaN_3_).

No interventions were performed on human participants or animals for this study. The teeth were extracted solely for orthodontic or periodontal reasons and voluntarily donated by informed patients for research. The study followed the Declaration of Helsinki and all ethical guidelines to protect the donors’ rights and well-being.

The selected root canals were categorized into three distinct age groups y: “young” (20–35 years), m: “middle-aged” (45–60 years), and o: “old” (70–85 years). To enhance the clarity of age-related comparisons, ten-year intervals between the groups were excluded. Ten samples from each age group (y, m, o) were assigned to one of the six luting systems under investigation (Table 1), creating a total of 18 groups with 10 samples each (n = 10 per group).

**Table 1:** Materials used in this study

Code	Material (batch no.)	Type	Manufacturer	Composition*
P21	Panavia 21 (000059)	Auto-cure, multi-step adhesive resin cement	Kuraray, Tokyo, Japan	10-MDP; hydrophobic aromatic and aliphatic as well as hydrophilic aliphatic dimethacrylate; silanized titanium dioxide, barium glass and silica filler; colloidal silicon dioxide (silica); catalysts; accelerators; pigments
	ED Primer	Auto-cure, self-etch adhesive	Kuraray, Tokyo, Japan	HEMA; 10-MDP; 5-NMSA; water; catalysts, accelerator
CXF	Core X Flow (180121)	Dual-cure, multi-step adhesive resin cement	Dentsply Sirona, Charlotte, NC USA	UDMA; di- & trifunctional methacrylates; barium boron fluoroaluminosilicate glass; camphorquinone; photoinitiator; photoaccelerators; silicon dioxide; benzoyl peroxide
	Prime&Bond active mit Self-Cure Activator	Dual-cure, universal adhesive	Dentsply Sirona, Charlotte, NC USA	Bisacrylamide, 2-propanol, 10-MDP, PENTA, camphorquinone, 4-dimethylaminobenzonitrile, UDMA, HEMA, catalysts, photoinitiators, stabilisers, acetone, water
ML	Multilink Automix (Y32283)	Dual-cure, multi-step adhesive resin cement	Ivoclar Vivadent, Schaan, Liechtenstein	Dimethacrylate, HEMA, barium glass, ytterbium trifluoride, spheroid mixed oxide, initiators, phosphonic acid, acrylic acid monomers
	Multilink Primer	Auto-cure, self-etch adhesive	Ivoclar Vivadent, Schaan, Liechtenstein	Initiators, HEMA, phosphonic acid and acrylic acid monomers
PSA	Panavia SA Cement Plus (2E0259)	Dual-cure, self-adhesive, one-step resin cement	Kuraray, Tokyo, Japan	10-MDP; Bis-GMA; TEGDMA; HEMA; hydrophobic aromatic and aliphatic dimethacrylate; silanized barium glass filler and colloidal silicon dioxide; surface-treated sodium fluoride; dl-camphor quinone, benzoyl peroxide; catalysts; accelerator; pigments
SM2	Smart Cem 2 (1806061)	Dual-cure, self-adhesive, one-step resin cement	Dentsply Sirona, Charlotte, NC USA	UDMA, EBPADMA, Di- and tri-functional function diluents, PENTA, 4-META, initiators, accelerators, stabilizer, barium boron fluoroaluminosilicate glass, amorphous silicon dioxide
SCP	SpeedCEM Plus (Y15127)	Dual-cure, self-adhesive, one-step resin cement	Ivoclar Vivadent, Schaan, Liechtenstein	UDMA, TEGDMA, PEGDMA, DDDMA, MDP, dibenzoyl peroxide, stabilizer, barium glass, silica ytterbium trifluoride
*Information provided by the manufacturer. 10-MDP: 10-Methacryloyloxy-decyl dihydrogen phosphate; HEMA: Hydroxyethyl methacrylate; 5-NMSA: N-methacryloyl-5-aminosalicylic acid; PENTA: dipentaerythritol pentacrylate phosphate; Bis-GMA: Bisphenol A diglycidyl methacrylate; TEGDMA: Triethylene glycol dimethacrylate; UDMA: Urethane dimethacrylate, EBPADMA: Ethoxylated Bis Phenol A Dimethacrylate; 4-META: Methacryloxyethyl trimellitate anhydride; PEGDMA: Polyethylene glycol dimethacrylate; DDDMA: 1,10-decandiol dimethacrylate

To ensure a consistent intracanal surface area of approximately 17.1 mm^2^ and to prevent the development of extra-axial lateral forces, all samples were horizontally decapitated to a working length (WL) of 8 mm. Root canal instrumentation was performed mechanically using the F360 file system (Komet Dental, Lemgo, Germany) with 0.04/#25 and 0.04/#35 files to the full WL of 8 mm. The canals were then further extended to a shortened length of 7.5 mm using a 0.04/#45 file to maintain congruence between the root canal and the post after instrumentation. Final preparation was completed with the BioRaCe7 system (FKG Dentaire Sàrl, La Chaux-de-Fonds, Switzerland) using a 60.02 file up to a WL of 8 mm. Throughout the root canal treatment, the canals were rinsed with distilled water (Sanismart, Waltrop, Germany) to eliminate any potential adverse effect from the rinsing process, ensuring reliable experimental conditions.

### Pull-out Test

To measure intracanal dentin adhesion, standardized, silica-coated, and silanized ISO 60 steel spreaders (Komet Dental, Lemgo, Germany) were employed in a modified pull-out test, following the method described by Ebert et al.^5^ After pretreating both the spreaders and root canals with adhesive according to the manufacturer’s instructions and applying the corresponding luting materials (Table 1), the spreaders were inserted into the canals to a WL of 8 mm. After a one-week storage period, the maximum bond strength (Fmax) was determined using a Zwick 1120 universal testing machine (Zwick Roell, Ulm, Germany) at a testing speed of 2 mm/min.

### Evaluation of Failure Mode

To assess the failure mode, fracture patterns were visually analyzed using a digital SLR camera, Canon EOS 500D (Canon, Tokyo, Japan), fitted with a microlens (Canon MP-E 65) at 3× magnification, capturing images from both sides. The failure modes were classified as follows: adhesive failure to the dentin (AD), adhesive failure to the spreader (AS), cohesive failure within the luting composite (C), and mixed failure (M).

### Statistical Analysis

The collected data were analyzed statistically using SPSS 23 for Mac (IBM, Armonk, NY, USA) and R version 4.2.1 (R Foundation for Statistical Computing, Vienna, Austria).^17^ Intracanal dentin adhesion was compared across three age groups and six luting systems via a non-parametric two-factor ANOVA. Since the age factor and the interaction between age and luting systems were not significant, Tukey’s post-hoc test was applied for all pairwise comparisons of the luting systems. The non-parametric ANOVA was conducted following the methods outlined in Brunner et al^3^ on p. 263, utilizing the R package rank FD version 0.0.1. Although the confirmatory statistical analysis utilized non-parametric methods, the medians and interquartile ranges (IQR), as well as means and standard deviations (SD) were reported for all metric variables within each group and provided separately for the material groups. Additionally, a Chi-square test assessed the association between bond strength and fracture mode. The significance level for the primary analysis (non-parametric two-factor ANOVA) was set at α = 0.05. Sample size determination through power analysis was not conducted prior to the study. However, assuming a sample size of n = 10 per group, the two-sample *t*-test achieves a power of 80% with a two-sided α = 0.05 and an effect size of d = 1.33. Since age did not significantly affect dentin adhesion, nor was there an interaction between luting systems and age, the age groups were pooled to yield a sample size of n = 30 for each luting system. Consequently, Tukey’s post-hoc test could detect clinically relevant differences between the luting systems.

## RESULTS

The position and dispersion of the data obtained were shown in Table 2 for all 18 groups (n = 10) and in Table 3 for the individual materials (n = 30). Patient age had no significant impact on bond strength (ANOVA; P = 0.506), nor was there a significant interaction between the luting system and age (ANOVA; P = 0.382). In contrast, bond strength varied significantly among the materials tested (ANOVA; P < 0.001), with further analysis using Tukey’s post-hoc test, as detailed in Table 4.

**Table 2:** Bond strength medians (IQR) and mean values ± SD [MPa] for all experimental groups

Experimental groups	P21	CXF	ML	PSA	SM2	SCP
y	Median (IQR) Mean ± SD	5.39 (4.52) 5.56 ± 2.74	9.31 (5.17) 9.45 ± 2.74	8.71 (1.60) 8.56 ± 1.04	3.48 (4.63) 3.84 ± 3.82	8.90 (3.56) 9.54 ± 2.40	6.48 (7.83) 7.24 ± 3.65
m	Median (IQR) Mean ± SD	7.16 (3.44) 7.33 ± 2.74	7.51 (5.19) 6.55 ± 2.92	7.64 (4.28) 7.28 ± 2.14	3.14 (3.33) 3.58 ± 2.38	10.30 (3.90) 9.59 ± 2.41	7.31 (2.03) 7.15 ± 1.30
o	Median (IQR) Mean ± SD	5.68 (4.29) 8.32 ± 6.67	7.96 (5.02) 7.67 ± 2.90	8.07 (1.12) 7.77 ± 1.53	6.23 (2.88) 6.31 ± 2.62	8.91 (3.19) 9.36 ± 3.24	7.95 (2.59) 7.55 ± 2.35
P21: Panavia 21 with ED Primer, CXF: Core X Flow with Prime&Bond active and Self-Cure Activator, ML: Multilink Automix with Multilink Primer, PSA: Panavia SA Cement Plus, SM2: Smart Cem 2, SCP: Speed CEM Plus, y: young (20–35 years), m: middle-aged (45–60 years), o: old (70–85 years)

**Table 3:** Bond strength medians (IQR) and mean values ± SD [MPa] for luting materials

Luting material	P21	CXF	ML	PSA	SM2	SCP
Median (IQR)	5.86 (3.42)	8.07 (3.96)	8.08 (1.77)	4.18 (3.90)	9.04 (3.35)	7.32 (3.05)
Mean ± SD	7.07 ± 4.45	7.89 ± 3.01	7.87 ± 1.67	4.58 ± 3.16	9.50 ± 2.62	7.31 ± 2.53


**Table 4:** Pairwise comparisons of luting materials according to Tukey’s post-hoc test

Compared materials	PSA vs P21	PSA vs SM2	PSA vs CXF	PSA vs SCP	PSA vs ML	P21 vs SM2	P21 vs CXF	P21 vs SCP	P21 vs ML	SM2 vs CXF	SM2 vs SCP	SM2 vs ML	CXF vs SCP	CXF vs ML	SCP vs ML
p-value	0.193	<0.001	0.003	0.021	<0.001	0.003	0.419	0.926	0.169	0.290	0.023	0.234	0.909	1.000	0.697


**Fig 1:**
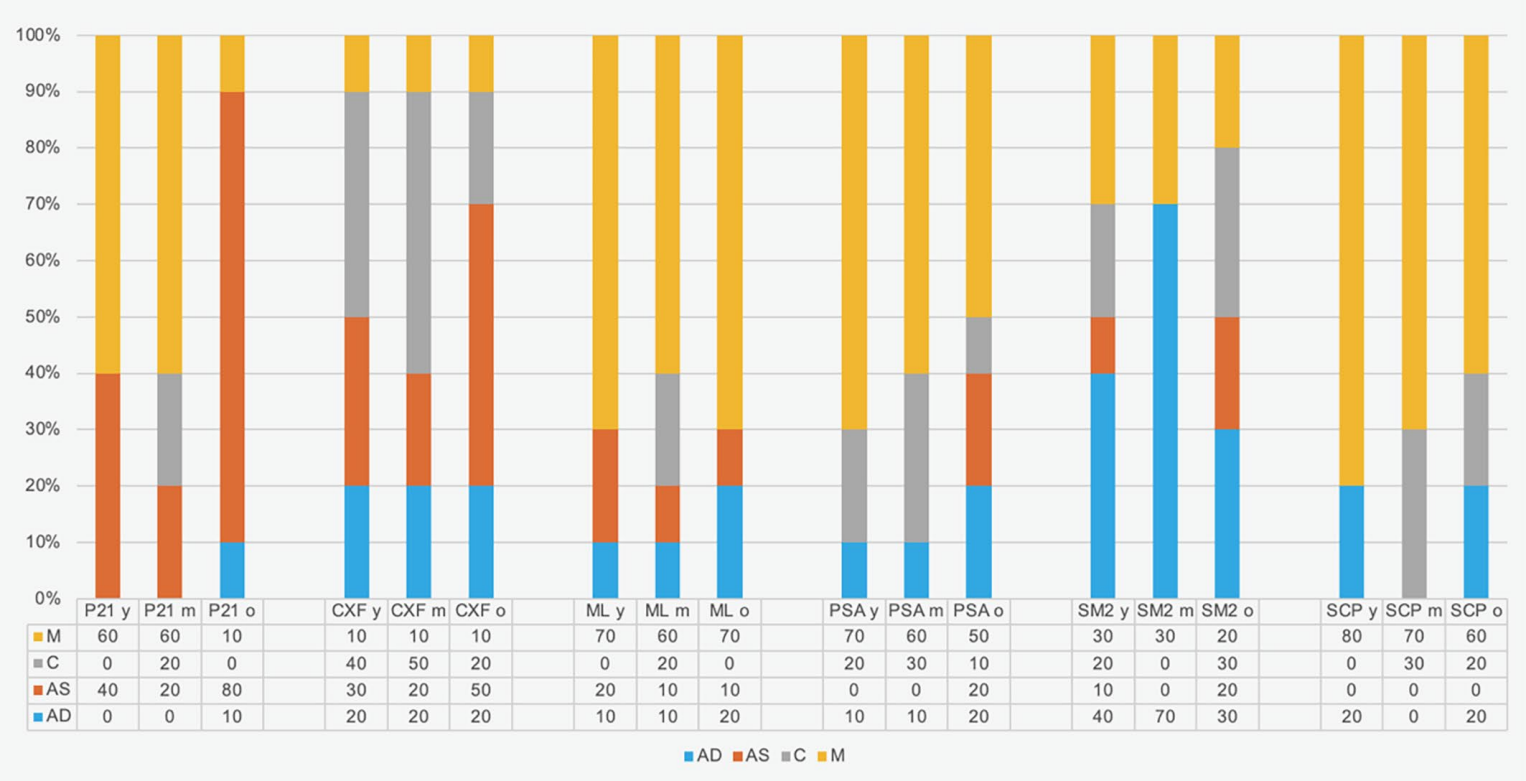
Failure mode distribution (%) in luting systems and in patient age. P21: Panavia 21 with ED Primer, CXF: Core X Flow with Prime&Bond active and Self-Cure Activator, ML: Multilink Automix with Multilink Primer, PSA: Panavia SA Cement Plus, SM2: Smart Cem 2, SCP: Speed Cem Plus, y: young (20–35 years), m: middle-aged (45–60 years), o: older (70–85 years), M: mixed failure, C: cohesive failure, AS: adhesive failure to spreader, AD: adhesive failure to dentin.

The distribution of fracture modes is shown in Figure 1. Fracture mode M was the most common, occurring in 46% of cases, with a frequency of 53% in group y and 70% in SCP. Fracture mode AS was most prevalent in group o (30%) and in P21 (47%). Significant differences in fracture modes were found both among the luting materials (Chi-square; P < 0.001) and in relation to patient age (Chi-square; P = 0.042).

## DISCUSSION

Based on the presented results, the null hypothesis – that there are no differences in intracanal dentin adhesion – was rejected for the luting systems but confirmed for the age groups. While the “old” group and P21 samples exhibited a higher frequency of adhesive failure to the spreader (AS) (Fig 1), the study did not reveal a consistent improvement in intracanal bond strength either across different age groups or between one-step (PSA, SM2, SCP) and multistep adhesive resin cements (P21, CFX, ML).

The age-related mineralization process in dentin, driven by heterogeneous nucleation, remains incompletely understood. Intra-tubular hydroxyapatite precipitates^16^ form smaller crystallites than those found in intertubular dentin.^8^ These are either passively precipitated from the intertubular matrix^16^ or grow centripetally on the inner walls of peritubular dentin.^1^ The resulting intra-tubular mineral phase resembles peritubular dentin in its high radiopacity and texture^15^ but lacks the proteolipid-phospholipid concentration^7^ and collagen matrix typical of intertubular dentin.^24^

With aging, intertubular collagen undergoes maturation, leading to a more cross-linked collagen network.^9, 13^ The collagen fibrils transform into fiber bundles,^23^ although their distribution and content remain unchanged.^25^ Factors such as aging, disease, and elevated blood glucose levels^12^ result in the formation of advanced glycation end products (AGEs), which accumulate in the dentin’s organic matrix and contribute to changes in the collagen structure.^19^

Despite the age-related alterations in the dentin’s apatite and collagen phases, as well as their mechanical^26^ and physical effects,^20^ this study did not observe significant changes in bond strength with tooth age when comparing one-step and multistep adhesive resin cements. Increased bond strengths appear to depend on the chemical composition of the luting material rather than on the natural conditions of dentin.

In particular, the functional monomer 10-MDP, known to enhance the bond strength of P21^11^ and PSA,^18^ appeared to be less effective as the sole primary active ingredient when compared to other materials. This suggests that, while 10-MDP plays a critical role in adhesion, its performance may be improved or complemented by other monomers or agents in different formulations.

However, these results could vary in clinical settings where endodontic irrigants that remove the smear layer might influence the adhesion of self-etching materials.^2^ Additionally, the histological dentin structure may vary due to individual physiological wear.^8^ Clinically, detachment of the adhesive bond is not solely influenced by axial forces. To obtain more meaningful results, future studies should consider additional factors affecting adhesion in the oral cavity, such as temperature fluctuations, chewing forces, and chemical or enzyme attacks, alongside larger sample sizes.

## CONCLUSION

Based on the present results, no age-related differences in bond strength were observed in this study. This suggests that all the systems tested are equally suitable for bonding root posts in teeth across all age groups (young, middle-aged, and older patients). Significant variations in adhesive bond strength were found only between the different luting systems, and these should be considered in clinical practice when selecting materials for adhesive luting.

### Clinical Relevance

The findings of this study provide valuable insights that can enhance the scientific foundation for selecting and applying adhesive and restorative procedures, particularly in relation to patient age. This knowledge could help reduce clinical failures and improve the overall prognosis of treatment methods.
